# Gemcitabine treatment promotes immunosuppressive microenvironment in pancreatic tumors by supporting the infiltration, growth, and polarization of macrophages

**DOI:** 10.1038/s41598-018-30437-2

**Published:** 2018-08-10

**Authors:** Sachin Kumar Deshmukh, Nikhil Tyagi, Mohammad Aslam Khan, Sanjeev Kumar Srivastava, Ahmed Al-Ghadhban, Kari Dugger, James Elliot Carter, Seema Singh, Ajay Pratap Singh

**Affiliations:** 1 Department of Oncologic Sciences, Mitchell Cancer Institute, University of South Alabama, Mobile, AL 36604 USA; 2 Division of Cell Biology and Genetics, Tatva Biosciences, Coastal Innovation Hub, 600 Clinic Drive, 3rd Floor, Mobile, AL 36688 USA; 3 Department of Clinical and Diagnostic Sciences, University of Alabama at Birmingham, Birmingham, AL 35294 USA; 4 Department of Pathology, College of Medicine, University of South Alabama, Mobile, AL 36617 USA; 5 Department of Biochemistry and Molecular Biology, College of Medicine, University of South Alabama, Mobile, AL 36688 USA

## Abstract

Chemotherapy-induced immunosuppression poses an additional challenge to its limited efficacy in pancreatic cancer (PC). Here we investigated the effect of gemcitabine on macrophages, which are the first line of immune-defense mechanisms. We observed an increased presence of macrophages in orthotopic human pancreatic tumor xenografts from mice treated with gemcitabine as compared to those from vehicle only-treated mice. Conditioned media from gemcitabine-treated PC cells (Gem-CM) promoted growth, migration and invasion of RAW264.7 macrophage. In addition, Gem-CM also induced upregulation of M2-polarized macrophage markers, arginase-1 and TGF-β1. Cytokine profiling of gemcitabine-treated PC cells identified IL-8 as the most differentially-expressed cytokine. Incubation of Gem-CM with IL-8 neutralizing antibody diminished its ability to induce growth, migration and invasion of RAW264.7 macrophages, but did not abrogate their M2 polarization. Together, our findings identify IL-8 as an important mediator in the gemcitabine-induced infiltration of macrophages within the pancreatic tumor microenvironment and suggest the requirement of additional mechanism(s) for macrophage polarization.

## Introduction

Pancreatic cancer (PC) is the third leading cause of cancer-related death in the United States, and remains one of those cancers that have seen no significant improvements in their clinical outcome over past several decades^1,2^. More upsettingly, it is expected to become the second leading cause of cancer-related death by the year 2030 or even earlier considering the continued increases in its incidence and mortality^3^. According to the American Cancer Society, approximately 55,440 patients are expected to be diagnosed with PC this year and about 44,330 people will succumb to this disease^4^. Gemcitabine, a nucleoside analogue, is used either as a single agent or in combination with other chemotherapeutic agents to treat PC, but these therapies provide marginal benefits only to the PC patients^5^. The poor outcomes of current therapies are largely associated with inherent or acquired chemoresistance of PC cells^6–8^. Furthermore, unique properties of pancreatic tumor microenvironment (TME) are also believed to play an important role in the unusual chemoresistance of PC^9–11^.

Regardless of their curative efficacy, most chemotherapies are associated with wide range of adverse effects on non-target tissues. Chemotherapeutic treatment is associated with a significant negative impact on the immune system including increased recruitment of the tumor supportive immune cells in the TME. More importantly, in the context of PC, tumor-infiltrated or tumor-associated macrophages (TAMs) have been shown to promote cancer stemness and chemoresistance^12,13^. Therefore, the present study was undertaken to examine the effect of gemcitabine treatment on pancreatic tumor immune-microenvironment, especially on macrophages. Our data demonstrate that orthotopic human pancreatic tumor xenografts from gemcitabine-treated mice have greater infiltration of macrophages of the M2 phenotype. Further, our data show that the conditioned media from gemcitabine-treated human PC cells (MiaPaCa-2 and Colo-357) promotes migration, invasion, growth, and M2 polarization of RAW264.7 macrophages. Mechanistically, we have identified IL-8 to be a crucial factor in gemcitabine induced growth, migration and invasion of macrophages, but it did not appear to be involved in their M2 polarization. Together, these significant findings could be useful in developing approaches for better clinical management of PC by overcoming unintended immunosuppressive effect of chemotherapy.

## Results

### Gemcitabine-treated pancreatic tumors exhibit greater infiltration of macrophages with M2 phenotype

**Figure 1:**
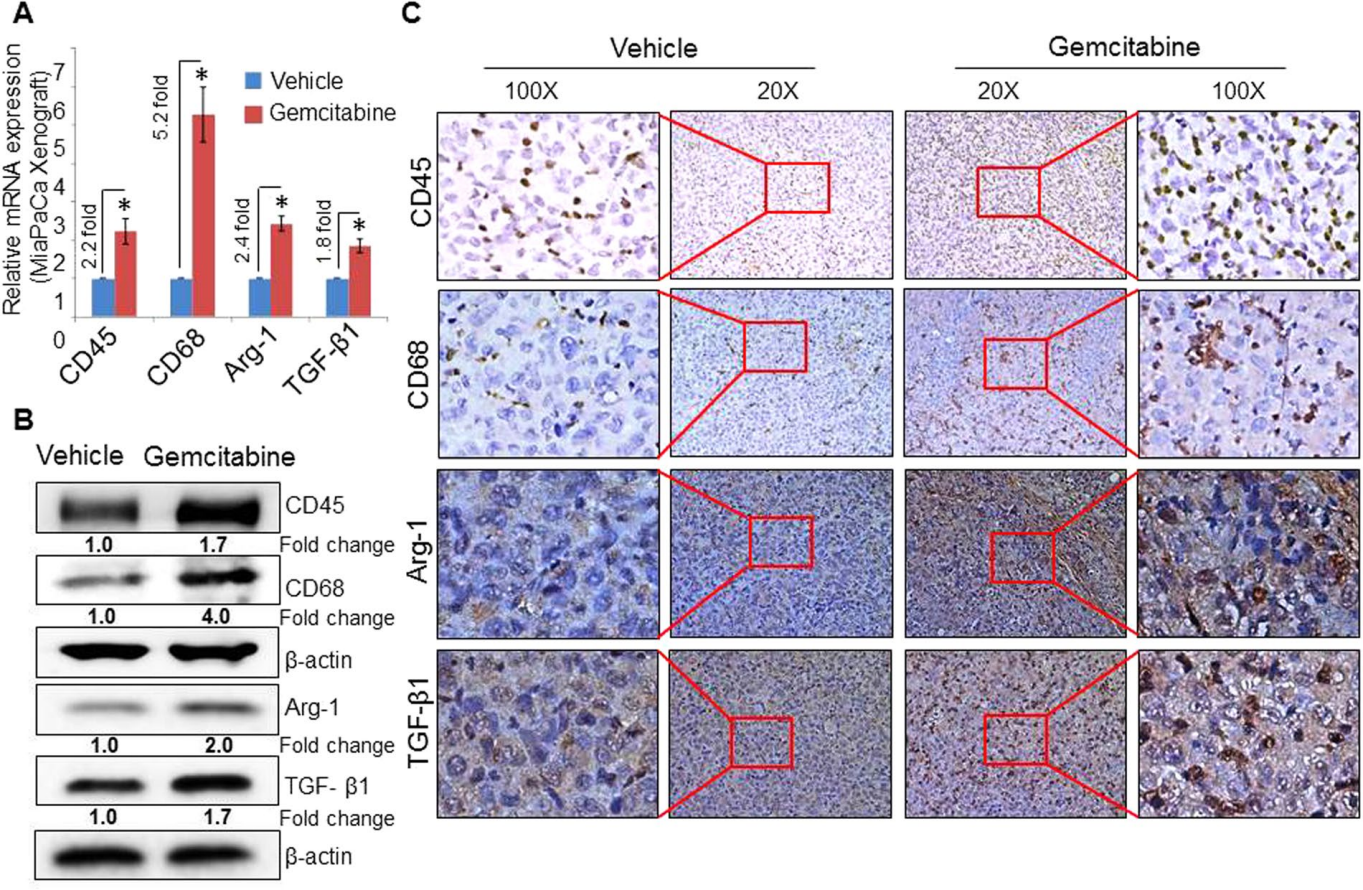
Gemcitabine induces a specific increase in macrophage infiltration in pancreatic tumors. (**A**) cDNA was prepared, and qRT-PCR was performed for transcripts of CD45 (all leukocytes), CD68 (macrophages), Arg-1 and TGF-β1 using the total RNA from tumor xenografts of either vehicle or gemcitabine-treated mice. GAPDH was used as internal control. Bars represent mean ± SD. *p < 0.05. (**B**) Western blot analyses of whole-tumor lysate to analyze the expression of CD45, CD68, Arg-1 and TGF-β1 protein detection. β-actin was used as an internal control. Fold change indicates the level of expression after normalization with β-actin. (**C**) Representative images (20X and 100X) of tumor sections (tumors were resected from mice treated with vehicle or gemcitabine) that were stained with either CD45, CD68, Arg-1 or TGF-β1.

To examine the effect of chemotherapy on immune microenvironment, we studied orthotopically-grown pancreatic tumors from either vehicle- or gemcitabine-treated mice. Total RNA and protein were isolated from frozen pancreatic tumor xenografts, and expression of immune cell-specific biomarkers was examined. Our data from the RT-PCR analysis showed an elevated expression of the common leukocyte marker, CD45 (2.2-fold) and CD68 macrophage marker (5.2-fold) in xenograft tumors from gemcitabine-treated mice as compared to vehicle treated group (Fig. 1A). We next examined the expression of Arg-1 and TGF-β1, classical markers of the M2 phenotype of macrophages, and observed their elevated levels in gemcitabine-treated tumor tissues (Fig. 1A). Consistent to this, we also observed enhanced expression of CD45, CD68, Arg-1 and TGF-β1 at the protein level as evident by our immunoblot analyses (Fig. 1B). We subsequently conducted immunohistochemical analyses on formalin-fixed tumor slices and recorded an increased presence of CD45^+^/ CD68^+^ cells having an elevated expression of Arg-1 and TGF-β1 in tumors from gemcitabine-treated mice, compared to those treated with vehicle only (Fig. 1C). We also analyzed pancreatic tumor sections for F4/80, a marker specific for mouse macrophages by immunohistochemistry staining. Increased staining of F4/80^+^cells was observed in tumor sections from gemcitabine-treated mice as compared to those of vehicle-treated mice (Supplementary Fig. 1). Together, these findings suggest that gemcitabine treatment triggers an increased infiltration of immune cells, specifically, M2 macrophages in pancreatic tumors.

### Effect of conditioned media from gemcitabine-treated pancreatic cancer cells on the migration and invasiveness of the macrophages

**Figure 2:**
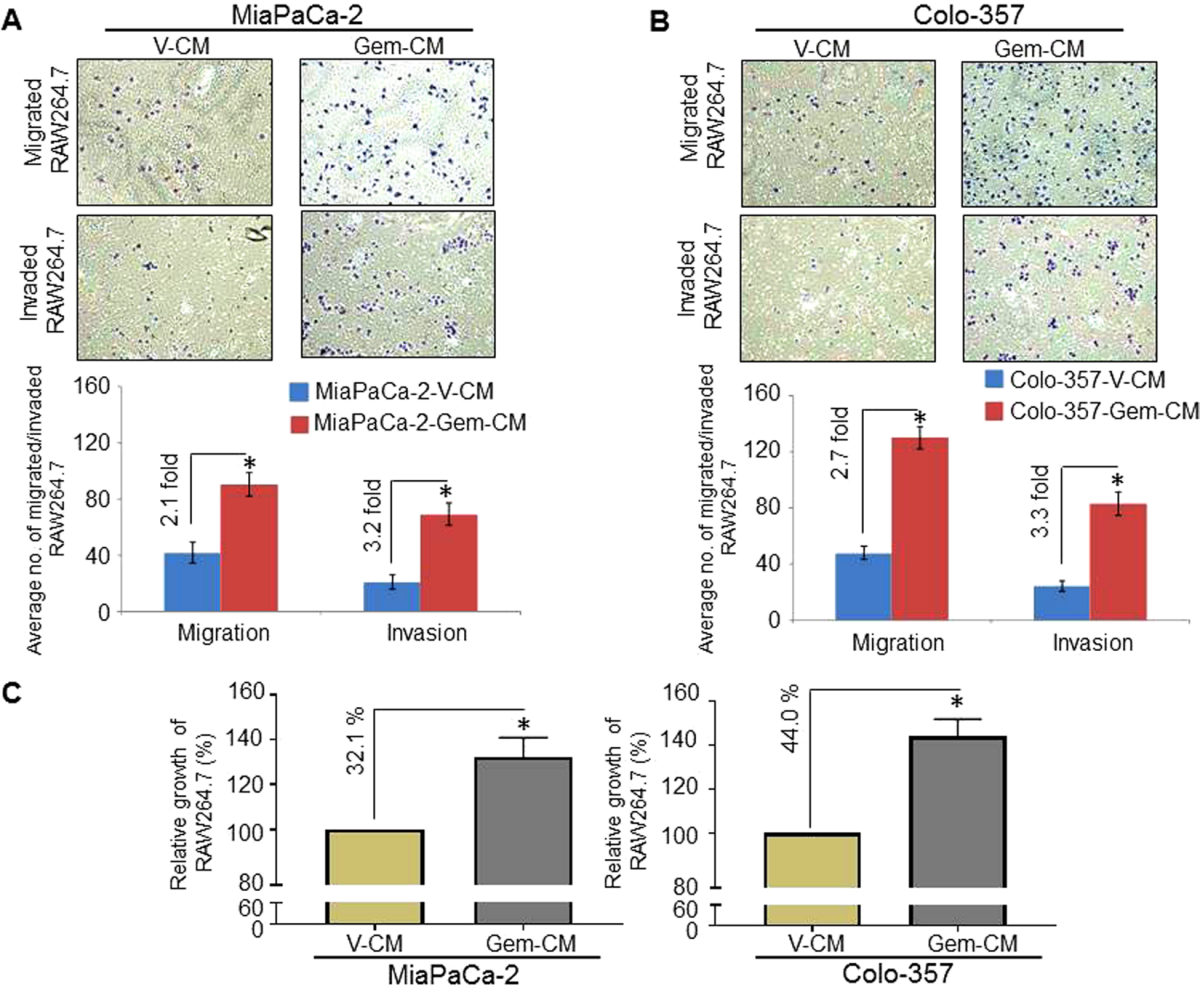
Conditioned media from gemcitabine-treated pancreatic cancer cell lines promotes motility, invasion, and growth of RAW264.7 macrophages. RAW264.7 macrophages were seeded on non-coated (2.5 × 10^5^/well for motility assay), or Matrigel-coated (5 × 10^4^/well for invasion assay) membranes. Conditioned media (CM) obtained from either vehicle (V-CM), or gemcitabine (Gem-CM) treated PC cell line cultures (**A**) MiaPaCa-2 or (**B**) Colo-357 were used as a chemoattractant in the lower wells. Migrated and invaded cells were counted in 10 random fields and presented as an average number of cells in 10 random field ± SD. (**C**) RAW264.7 macrophages (5 × 10^4^ cells/well) were seeded into 96-well plates. After growth for 24 h, cells were treated with V-CM or Gem-CM. The growth of RAW264.7 was measured by a WST-1 assay after 48 h of incubation. Data (mean ± SD, n = 3) shown as change in growth as compared to control. *p < 0.05.

Because we observed the increased infiltration of macrophages in gemcitabine-treated tumor sections, we investigated if it was due to the influence of gemcitabine on pancreatic tumor cells causing a release of immune-modulatory secretory signals. For this, we collected conditioned media from vehicle- (V-CM) and gemcitabine-treated (Gem-CM) pancreatic cancer cells (MiaPaCa-2 and Colo-357) and examined their chemo-attractive potential towards macrophages. Mouse macrophages (RAW264.7) were seeded into the top chamber of either a non-coated or Matrigel-coated membrane insert in serum-free media. The lower chamber of transwell plate contained either V-CM or Gem-CM to serve as chemoattractants. RAW264.7 macrophages were allowed to migrate or invade through the transwell membrane for 16 h and subsequently fixed and stained. Quantitation of stained macrophages on the lower surfaces of non-coated transwell membranes in random fields demonstrated a significant increase in RAW264.7 migration (~2.1- and ~2.7-folds, respectively) when Gem-CM obtained from MiaPaCa-2 and Colo-357 was used as the lower chamber chemoattractant as compared to V-CM (Fig. 2A,B). Similarly, macrophages on the lower surfaces of Matrigel-coated transwell membranes showed ~3.2, and ~3.3 fold increased invasion when Gem-CM was used as a chemoattractant as compared to V-CM from MiaPaCa-2 and Colo-357 respectively (Fig. 2A,B).

### Gem-CM enhances the growth of RAW264.7 macropha**ges**

Having observed the effect of Gem-CM on enhanced migration and invasion of RAW264.7 macrophages, we next examined whether Gem-CM had any effect on the growth of RAW264.7 macrophages. For these studies, RAW264.7 cells were seeded into a 96-well plate in the presence of either V-CM or Gem-CM. After 48 h, the effect on the RAW264.7 growth was examined by a WST assay. Our data demonstrated that Gem-CM from MiaPaCa-2 or Colo-357 cultures induced significant growth of RAW264.7 cells (32.1% and 44.0%, respectively) as compared to the media collected from vehicle-treated cells (Fig. 2C). These findings suggest that Gem-CM has the potential to trigger the growth of RAW264.7 macrophages.

### Conditioned media from gemcitabine-treated pancreatic cancer cells induces differentiation of macrophages to M2 phenotype

**Figure 3:**
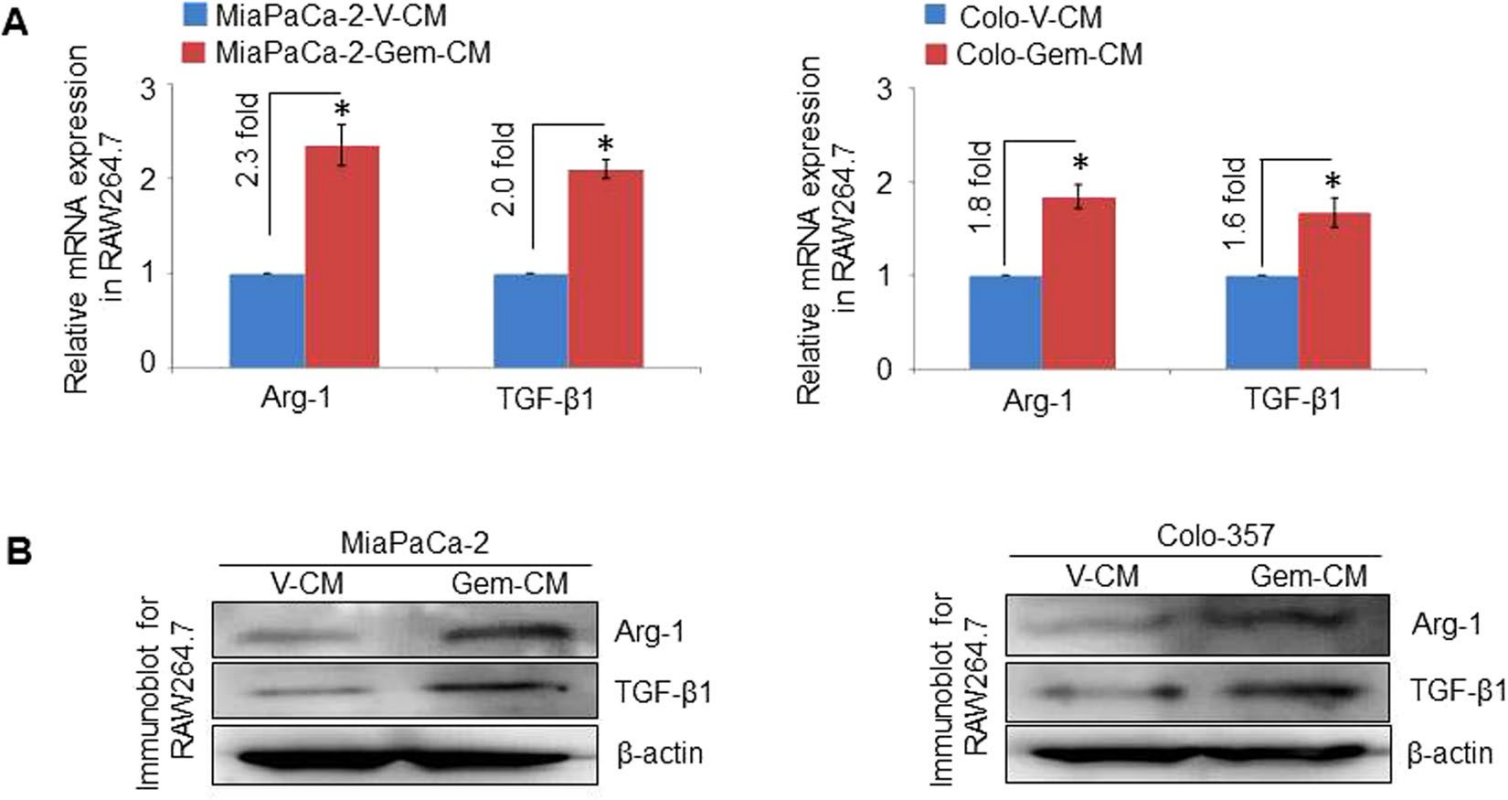
Conditioned media from gemcitabine-treated pancreatic cancer cell lines promotes M2 macrophage polarization. RAW 264.7 cells treated with either conditioned media from the vehicle (V-CM) or gemcitabine (Gem-CM) treated PC cell line cultures for 48 h. Total RNA was isolated, and cDNA was prepared to asses for expression of Arg-1 and TGF-β1, specific markers M2 macrophage polarization by qRT-PCR (**A**) and by western blot (**B**). Bars represent mean ± SD. *p < 0.05. GAPDH and β-actin were used as internal controls, respectively.

It has been suggested that M1 macrophages have anti-tumorigenic functions, while M2 macrophages exert pro-tumorigenic effects^14^. Since we observed greater infiltration of mouse macrophages of M2 phenotype in tumors resected from gemcitabine-treated mice, we next examined the effect of conditioned media derived from vehicle- and gemcitabine-treated pancreatic cancer cells (MiaPaCa-2 or Colo-357) on the polarization of RAW264.7 macrophages. We treated RAW264.7 cells with V-CM or Gem-CM for 48 h, and total RNA and protein were isolated. Subsequently, we examined the expression of M2 macrophage markers, Arg-1 and TGF-β1, by RT-PCR and immunoblot analyses. Significantly greater induction of the Arg-1 and TGF-β1 was detected in RAW264.7 cells, compared to V-CM at both the mRNA (Fig. 3A) and protein levels (Fig. 3B) suggesting that Gem-CM effectively induces M2 polarization of macrophages.

### Profiling of gemcitabine-treated pancreatic cancer cells identifies differential expression of various immunomodulatory cytokines

**Figure 4:**
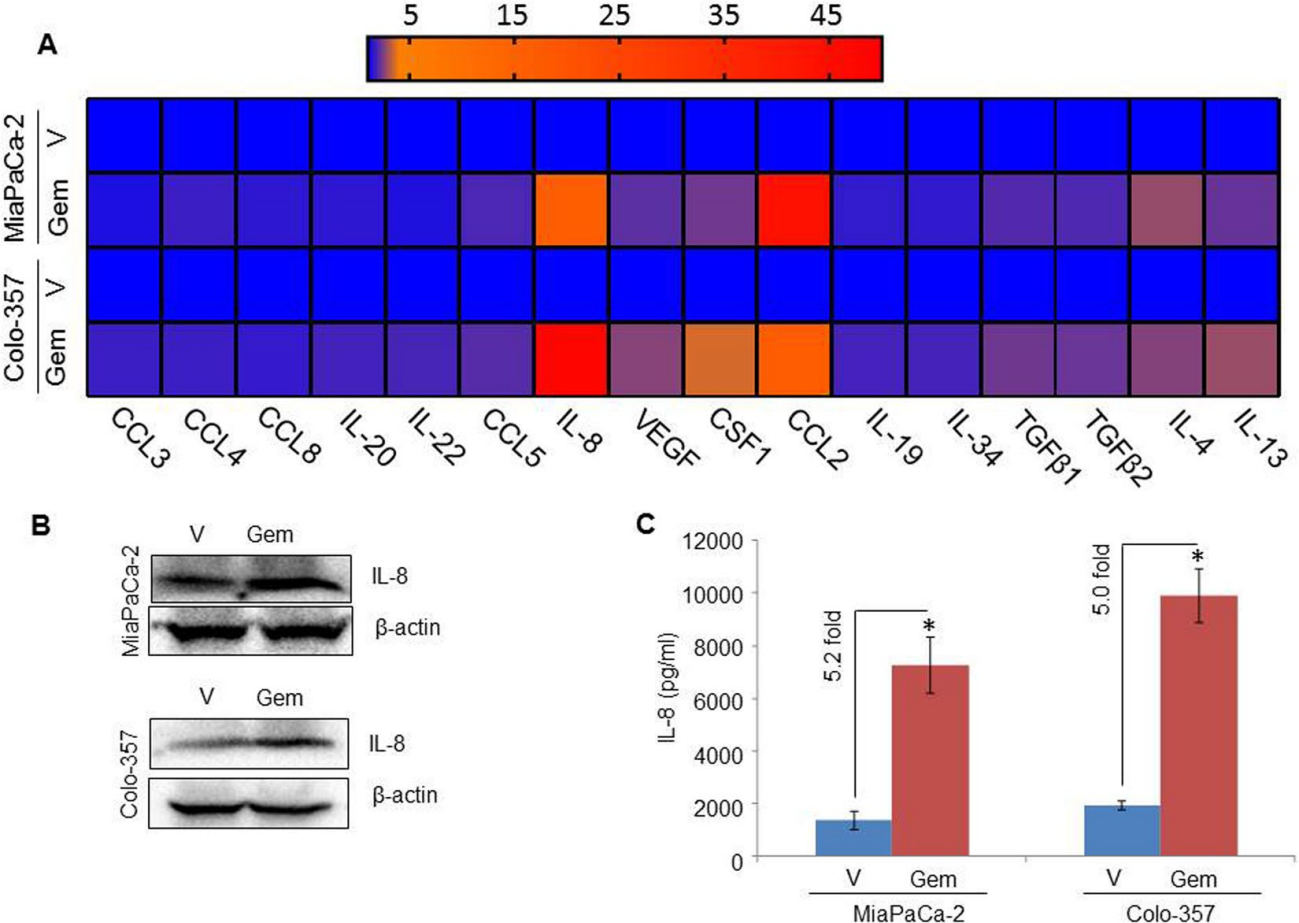
Gemcitabine induces expression of cytokines in pancreatic cancer cell lines. (**A**) Pancreatic cancer cell lines (MiaPaCa-2 or Colo-357) were treated with gemcitabine (10 µM) for 8 h. Subsequently, RNA was isolated, cDNA was prepared, and the expression of various cytokine genes was analyzed using qRT-PCR. (**B**) PC cell lines were treated with gemcitabine (10 μM) for 8 h. Post-treatment, media was replaced with fresh culture medium and incubated for another 24 h. Upon completion, total protein was isolated for immunoblot analysis of IL-8 expression. β-actin was used as a loading control. (**C**) The level of IL-8 in conditioned media of either vehicle or gemcitabine-treated PC cell lines was measured using a standard ELISA. Bars represent mean ± SD. n = 3, *p < 0.05.

Pancreatic tumor cells secrete cytokines that play a crucial role in immune modulation including the effect of macrophage infiltration, growth and polarization^14–16^. To examine the effect of gemcitabine treatment on the expression of immunomodulatory cytokines, we treated PC cells (MiaPaCa-2 or Colo-357) with either vehicle or gemcitabine for 12 h, and isolated total RNA from the treated cells. Subsequently, we profiled the transcript levels of cytokines and/or growth factors that have been associated with macrophage infiltration, growth or M2 polarization by performing quantitative RT-PCR analyses. We observed elevated levels of several cytokines and growth factors in gemcitabine-treated PC cell lines (Fig. 4A). Since the levels of IL-8 and CCL2 transcripts were found to be the most significantly elevated (~16 and ~47.9, and ~43.7 and ~16.9-folds, respectively in gemcitabine-treated MiaPaCa-2 and Colo-357), we examined their expression at protein levels by immunoblot and ELISA. Consistent with the RNA data, we observed enhanced expression of IL-8 at the protein level in both PC cell lines treated with gemcitabine (Fig. 4B); however, no expression of CCL2 was detected (data not shown) by western blot analysis. Our data from ELISA also demonstrated an increase in IL-8 in the culture supernatant of gemcitabine-treated MiaPaCa-2 and Colo-357 (Gem-CM) as compared to vehicle-treated cell cultures (Fig. 4C). Together, our data suggest that the treatment of gemcitabine induces the expression of various immunomodulatory cytokines by pancreatic cancer cells.

### Inflated IL-8 secretion by gemcitabine-treated pancreatic cancer cells mediates macrophage infiltration and growth, but not M2 polarization

**Figure 5:**
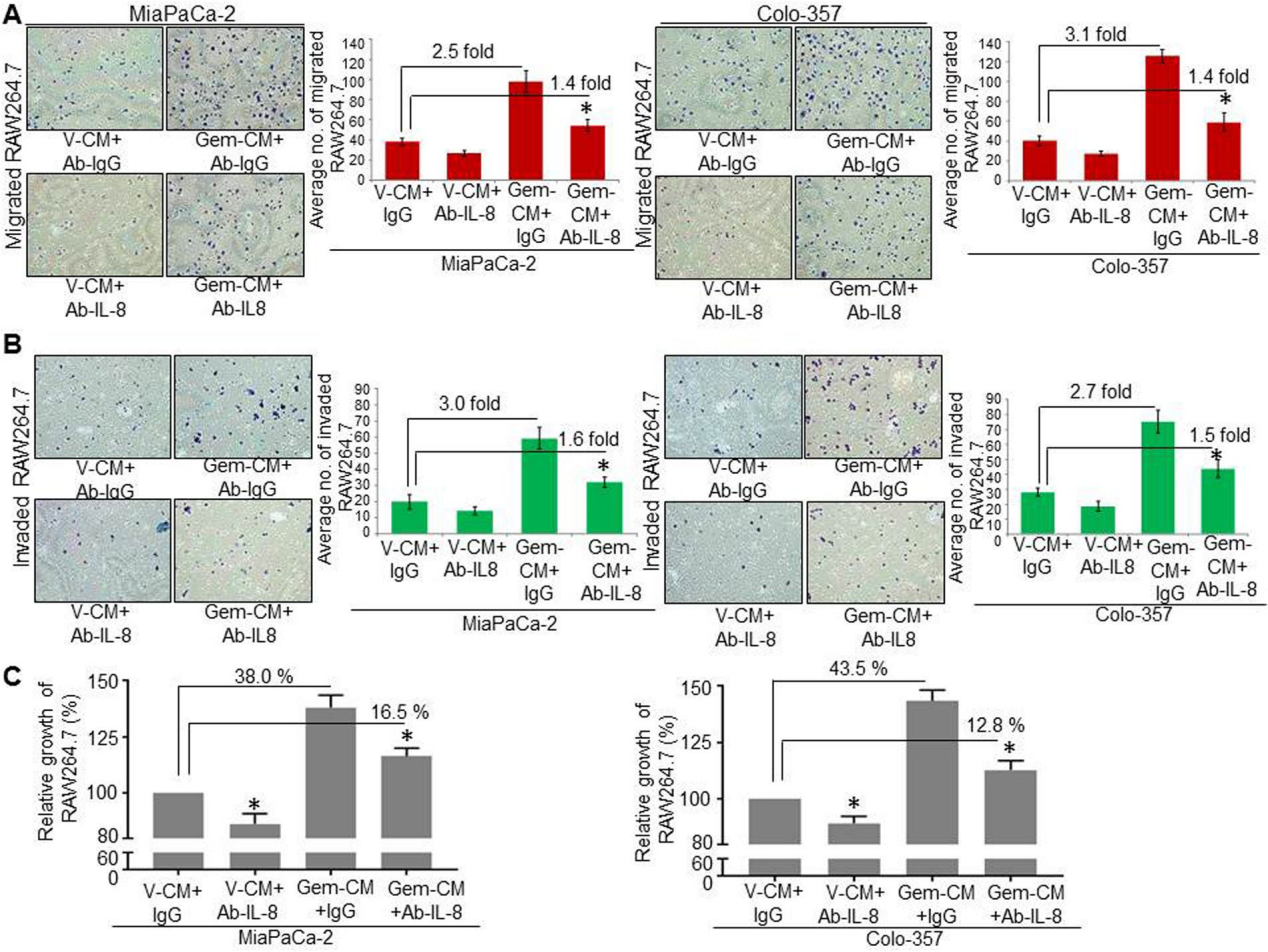
IL-8 neutralization within Gem-CM decreases the increased infiltration and growth of RAW264.7 macrophages. RAW264.7 cells were seeded on (**A**) non-coated (for motility assay), or (**B**) Matrigel-coated (for invasion assay) membranes, V-CM or Gem-CM pre-treated with IL-8 neutralizing antibody or control IgG (200 ng/mL) were used as a chemoattractant in the lower chambers. Bars represent mean ± SD of migrated or invaded cells per field. *p < 0.05. (**C**) RAW264.7 cells were seeded into a 96-well plate and treated with V-CM or Gem-CM pre-treated with IL-8 neutralizing antibody or control IgG (200 ng/mL) and growth was measured by a WST-1 assay after 48 h. Data (mean ± SD) shown as change in growth as compared to control. *p < 0.05.

Because we observed an induction of IL-8 following gemcitabine treatment of PC cell lines, we explored whether increased levels of IL-8 in Gem-CM promoted RAW264.7 macrophage migration, invasion, growth, and polarization. For these studies, V-CM and Gem-CM media from MiaPaCa-2 and Colo-357 cell cultures were pre-incubated with human IL-8 neutralizing antibody or control IgG for 24 h at 4 °C and subsequently centrifuged to obtain V-CM^dIL-8^ or Gem-CM^dIL-8^. Effect of V-CM^dIL-8^ or Gem-CM^dIL-8^ was assessed on RAW264.7 phenotypes. We observed that migration and invasion were significantly decreased in V-CM^dIL-8^ or Gem-CM^dIL-8^ treated RAW264.7 as compared to those treated with V-CM or Gem-CM (Fig. 5A,B). Similarly, we examined the effect of IL-8 neutralization on the growth of macrophages and observed a reduction in the macrophage growth upon neutralization of IL-8 in Gem-CM (Fig. 5C*)*. However, unlike the effect of IL-8 depletion on macrophage migration, invasion, and growth, we did not observe any effect of IL-8 neutralization on the expression of Arg-1 and TGF-β1 in RAW264.7 cells (Supplementary Fig. 2), suggesting that IL-8 is not a major mediator in the chemotherapy-induced M2 polarization of macrophages. To further ascertain the effect of IL-8 on macrophage migration and invasion, we seeded RAW264.7 on the top chamber of the non-coated or Matrigel-coated membrane and provided (10 ng/ml) recombinant IL-8 as a chemoattractant in the bottom chamber. Our observation revealed that in comparison to control, IL-8 significantly enhanced the migration (~3.1-fold) and invasion (~2.2-fold) of RAW264.7 macrophages (Supplementary Fig. 3). Similarly, we observed the growth potentiating the effect of IL-8 on macrophages, which is evident from our finding that IL-8 treatment enhanced the growth by ∼30% (Supplementary Fig. 4). Together, these findings confirm that gemcitabine triggers the expression of IL-8 in PC cells that induce the migration, invasion, and growth of macrophages.

## Discussion

This study explored the effect of gemcitabine treatment on the immune component of the pancreatic TME. Our findings provide experimental evidence of a greater accumulation of macrophages in orthotopic human pancreatic tumor xenografts from gemcitabine- treated mice relative to those from the vehicle-treated ones. *In vitro* studies demonstrated a greater potency of Gem-CM (conditioned-media from gemcitabine-treated PC cells) for induction of RAW264.7 macrophage growth, migration, invasion and polarization, compared to V-CM (conditioned-media from vehicle-treated PC cells). Moreover, we found IL-8 to be an important mediator in Gem-CM-induced growth and invasion, but not polarization, suggesting a mechanistic basis for our phenotypic observations. These are important findings as the growth and aggressiveness of tumor cells rely greatly on the TME due to bi-directional interactions between the tumor and non-transformed host cells.

High aggressiveness and adaptability to harsh surroundings of pancreatic tumor cells are likely due to their ability to rewire their molecular circuitry to establish a favorable connection with the host cells. Pancreatic TME is mostly comprised of activated pancreatic stellate cells/myofibroblasts, immune cells, endothelial cells and the proteins and other factors secreted by these cells. The bi-directional cross-talk between tumor and TME cells has been associated with the survival of pancreatic cancer cells under harsh environmental conditions, aggressive tumor phenotypes and chemoresistance^17^. The communication between the tumor cells and TME is regulated by a dynamic network of secreted cytokines, growth factors, and chemokines. These interactions result in remodeling of TME conditions, including an immunosuppressive microenvironment, that is conducive for tumor growth and sustenance^17^. In these contexts, our findings that the gemcitabine-treatment of pancreatic cancer cells can influence their pattern of immunomodulatory cytokines secretion are quite intriguing.

Infiltration and differentiation of macrophages to immunosuppressive phenotypes within TME has been associated with tumor progression and chemoresistance. Chemotherapeutic drugs have been associated with a toxicity that leads to immunosuppression that is manifested by the increased recruitment of tumor supportive immune cells, including Treg cells and M2 macrophages. Further, these immunosuppressive cells in the TME promote the depletion of anti-tumor lymphocyte populations^18,19^. Moreover, anti-cancer drugs hijack the immune cell effector functions and/or block their proliferative capacity, which leads to unchecked tumor growth^18^. Another dark side chemotherapy may possess is that it may trigger immunosuppression by inducing secretion of soluble mediators by cancer cells that result in the recruitment of additional immunosuppressive cells to the tumor site. Further, the polarization of these immune cells converts their actions to support tumor cells^18^. Aligned with these prior observations, our findings demonstrating enhanced recruitment of tumor supportive M2 polarized macrophages is highly significant and should help us in refining our treatment approaches for pancreatic cancer.

Macrophage migration, invasion, and growth can be regulated by multiple cues from cancer cells. In fact, recruitment of macrophages is tightly regulated by a fine balance of multiple factors that include cytokines, chemokines and/or growth factors present at the tumor site^20^. It has been suggested earlier that gemcitabine treatment programs PC cells for the secretion of angiogenesis promoting factors^21^. Additionally, our findings from this study clearly suggest that gemcitabine can induce the upregulation and expression of various growth factors/cytokines, including IL-8. Moreover, IL-8 was found to play a significant role in the Gem-CM-promoted effects on macrophages. IL-8 is a pro-inflammatory cytokine secreted by several cancer cells including PC cells^21,22^. IL-8 has been shown to positively affect the TME through autocrine and paracrine signaling^23^. IL-8 confers its biological effects by binding to two cell surface receptors, CXCR1 and CXCR2, which are widely expressed on macrophages^24,25^. It is well known to induce the infiltration of immune cells such as neutrophils and macrophages at the site of any damage or infection^26,27^. In RAW264.7 cells, IL-8 treatment is shown to cause induction of NF-ĸB activity^28^, which triggers the expression of matrix metalloproteinases and thus facilitate macrophage invasion^29^. However, IL-8 neutralization of Gem-CM had no effect on the polarization of macrophages to TAM suggesting that this phenotypic switch in response to gemcitabine-treatment requires the involvement of other factors, and thus warrants further investigations.

Macrophages are a plastic cell population that, according to environmental cues, can switch from an M0 to activated M1 state or to the immunosuppressive M2 state. Cancer cells recruit macrophages into the TME and promote their differentiation into TAMs that are equivalent to the M2 phenotype^30^. Clinicopathological studies argue that accumulation of TAM in tumors correlates with an increased adverse clinical outcome^31^. Emerging evidence from experimental and animal studies also suggest that TAMs provide fertile soil for tumor progression by releasing a diversity of cytokine, chemokine and growth factors that support tumor growth^14^. TAMs, directly and indirectly, affect the process of tumor metastasis by modulating TME^30^. TAM released immunosuppressive factors suppress the immune response of T cells which helps in tumor cell growth, survival and metastasis^32^. Moreover, TAMs produced chemokines mediate the trafficking of Treg to the tumor which counteracts T cell-mediated immune response^33,34^. The TAM secreted factors promote angiogenesis, matrix remodeling, and immune suppression that leads to increased therapy resistance and tumor progression^14,35^. The involvement of TAMs in cancer progression and therapy has only recently gained recognition^13^. Earlier it had been reported that PC cell lines, Panc-1 and BxPC-3, attain increased epithelial-mesenchymal transition (EMT) following co-cultures with M2-polarized TAMs^36^. Further, co-cultures of PC cells with TAMs increase proliferation, migration and proteolytic activity of PC cells^36^. Weizmen *et al*. suggest that TAMs can induce chemoresistance of PC cells by reducing gemcitabine-induced apoptosis^13^. Furthermore, in a preclinical model, authors have reported that reduced macrophage infiltration does improve tumor cell responses to gemcitabine^13^. Therefore, enhanced induction of M2 polarization of macrophages by secreted factors from gemcitabine-treated cells is a noteworthy finding that could not only have implications for chemoresistance, but can in fact further aggravate the resistant disease.

In conclusion, we have demonstrated that gemcitabine treatment of PC cells results in the induction of IL-8, which, in turn, promotes macrophage (RAW264.7) migration, invasion, and growth. Furthermore, Gem-CM obtained from PC cells also induces macrophage polarization to TAM/M2 phenotype in an IL-8-independent manner. These novel findings provide us the mechanistic insight into the immunosuppressive effects of gemcitabine, which could be helpful in the development of better therapeutic approaches for the treatment of pancreatic cancer.

## Materials and Methods

### Compliance with Ethical Standards

We obtained pancreatic tumor xenograft tissues from an animal study that was approved by the University of South Alabama Institutional Animal Care and Use Committee (IACUC). All the research methods were performed in accordance with the guidelines from IACUC and Institutional Biosafety Committee at the University of South Alabama.

### Cell lines and tissues

PC cell lines (MiaPaCa-2 and Colo-357) were procured and maintained as described earlier^37^. The mouse macrophage RAW264.7 cell line was procured from ATCC (Manassas, VA#TIB-71). All cell lines were maintained in Dulbecco’s Modified Eagle Medium (DMEM) containing L-glutamine, L-glucose, and sodium pyruvate, supplemented with 10% fetal bovine serum (FBS), penicillin (100 units/ml) and streptomycin (100 μg/ml) in a humidified atmosphere of 5% CO_2_ at 37 °C. All cell lines were regularly monitored for their typical morphology and intermittently tested for mycoplasma contamination at our Institutional facility. Pancreatic tumor tissue from the orthotopic xenograft mice treated with vehicle and gemcitabine were obtained from our ongoing study.

### Reagents and antibodies

The following reagents were used: Dulbecco’s Modified Eagle Medium (DMEM) (GE Healthcare Life Sciences, Logan, UT); Fetal Bovine Serum (FBS) (Atlanta Biologicals, Lawrenceville, GA); penicillin-streptomycin (Invitrogen, Carlsbad, CA); WST-1 proliferation assay kit (Roche, Indianapolis, IN); High-Capacity RNA-to-cDNA™ Kit and SYBR Green Master Mix (Applied Biosystems, Carlsbad, CA); Diff-Quick cell staining kit (Dade Behring, Inc., Newark, DE); anti-human IL-8 ELISA Kit (R&D Systems Inc., Minneapolis, MN); Gemcitabine (Sigma-Aldrich, St. Louis MO). Antibodies used were: anti-CD45, -CD68, -F4/80, -TGF-β1, -IL-8 (Abcam, Cambridge, MA), anti-arginase-1, anti-rabbit horseradish peroxidase (HRP)-conjugated secondary antibodies (Santa Cruz Biotechnology, Santa Cruz, CA) and anti-β-actin (Sigma-Aldrich). Western blotting SuperSignal West Femto Maximum sensitivity substrate kit was purchased from Thermo Scientific (Logan, UT). Immunohistochemical analysis reagent EZ-Dewax (Biogenex, Fremont, CA); background sniper, polymer, and probe were purchased from Biocare Medical (Concord, CA).

### RNA isolation and reverse transcription-polymerase chain reaction (RT-PCR)

Total RNA from the tumor tissue and cell line was extracted using the TRIzol reagent, and complementary DNA (cDNA) synthesized using 2 μg of total RNA and a High-Capacity RNA-to-cDNA™ Kit following the manufacturer’s (Applied Biosystems, Carlsbad) instructions. Quantitative real-time PCR (RT-PCR) was performed using cDNA and SYBR Green Master Mix on an iCycler system (Bio-Rad, Hercules, CA) using specific sets of primer pairs (Table S1). The thermal conditions for real-time PCR assays were as follows: cycle 1: 95 °C for 10 min, cycle 2 (×40): 95 °C for 10 sec and 58 °C for 45 sec. GAPDH was used as internal control.

### Immunohistochemical and histological analyses

Paraffin-fixed tumor tissues from the mice treated with vehicle or gemcitabine were processed for immunohistochemical analyses as described earlier^38^. Antibodies against CD45, CD68 F4/80 were used at a 1:200 dilution while Arg-1 and TGF-β1 were used at a 1:100 dilution.

### Collection of conditioned media

Pancreatic cancer cell lines, MiaPaCa-2 and Colo-357 cells (1 × 10^6^/well) were seeded in 6-well plates and allowed to grow to 60–70% confluency.Subsequently, cells were treated with either vehicle (PBS) or gemcitabine (10 μM) for 8 h. After treatment, cells were washed twice with PBS, and the media replaced with fresh low (2.5%) serum containing DMEM media. Cells were further allowed to grow for 48 h followed by a collection of the conditioned media (CM) from the vehicle or gemcitabine-treated cells. CM was centrifuged for 10 min at 2,500 rpm at 4 °C to remove the cell debris and designated as either V-CM (from vehicle-treated cells) or Gem-CM (from gemcitabine-treated cells).

### Immunoblot analysis

Total protein from tumor tissue, PC cell lines and RAW264.7 macrophages were isolated using NP-40 lysis buffer containing a protease phosphatase inhibitor cocktail. Thereafter, protein samples (60 µg) were resolved by SDS-PAGE and subjected to immunoblot analysis as described earlier^39^ using protein-specific antibodies. All primary antibodies were used at 1:1000 dilutions with a respective HRP labeled secondary antibody at a 1:2500 dilution. β-actin was used as an internal control at a 1:20,000 dilutions. The signal was detected with Super Signal West Femto maximum sensitivity substrate kit (Thermo Scientific, Logan, UT, USA) by using the ChemiDoc Imaging System (Bio-Rad).

### *In vitro* cell growth assay

To examine the effect of V-CM or Gem-CM on the growth of RAW264.7 cells (5 × 10^3^ cells/well) were seeded in a 96-well plate and cultured in complete DMEM media overnight. After 24 h, complete DMEM media was replaced with either V-CM or Gem-CM media (collected from vehicle- or gemcitabine-treated MiaPaCa-2 and Colo-357 PC cell lines). RAW264.7 cells were allowed to grow for 48 h. Cell growth was monitored by a WST-1 assay (Roche Diagnostics, Mannheim, Germany) as discussed previously^40^. To study the role of IL-8, Gem-CM (conditioned media derived from gemcitabine treatment) was incubated with either control IgG (200 ng/mL) or IL-8 neutralizing antibody (200 ng/mL) for 24 h at 4 °C. Antibody-treated Gem-CM media was centrifuged to remove any IL-8 bound antibody prior to RAW264.7 macrophage treatment.

### Migration and invasion assays

To analyze the effects of the either V-CM or Gem-CM on the migration and invasion, RAW264.7 cells were plated on the top chamber of either a non-coated polyethylene teraphthalate membrane (2.5 × 10^5^ cells/6-well plate inserts, for migration assay) or a Matrigel-coated polycarbonate membrane (1 × 10^5^ cells/24-well plate inserts, for invasion assay). Conditioned media (V-CM or Gem-CM) was added to the lower chambers to serve as a chemo-attractant. These experiments were performed as described previously^21^. To establish the role of IL-8 on migration and invasion of macrophages, we depleted IL-8 from either V-CM or Gem-CM by incubating the conditioned media with either control IgG or IL-8 neutralizing antibody (200 ng/mL) for 24 h at 4 °C. Subsequently, IL-8-depleted conditioned media was added to the lower chambers and used as the chemoattractant for both the migration and invasion assays.

### Enzyme-linked immunosorbent assay (ELISA)

ELISA was performed to measure the level of the IL-8 present in the conditioned media obtained from vehicle- or gemcitabine-treated PC cell lines as per manufacturer’s instructions.

### Statistical analysis

Wherever suitable the experiments were performed three times. The data were also subjected to unpaired two-tailed Student’s t-test wherever appropriate and p < 0.05 was considered statistically significant.

### Compliance with Ethical Standards

Studies involving the use of animals and cell lines were approved by the University of South Alabama Institutional Animal Care and Use Committee (IACUC) and Institutional Biosafety Committee respectively.

## Electronic supplementary material

Supplementary Information

## Data Availability

We confirm that all the data in this manuscript is original and we have access to the raw data files.
