# Supplementary material for: Gemcitabine treatment promotes immunosuppressive microenvironment in pancreatic tumors by supporting the infiltration, growth, and polarization of macrophages
Source: Sci Rep. 2018 Aug 10;8:12000. doi: 10.1038/s41598-018-30437-2 (PMC6086900; doi:10.1038/s41598-018-30437-2)
Supplement: Supplementary file 1 — Supplementary Information [file 41598_2018_30437_MOESM1_ESM.pdf]

Gemcitabine treatment promotes immunosuppressive microenvironment in pancreatic tumors by supporting the infiltration, growth, and polarization of macrophages

Sachin Kumar Deshmukh[1], Nikhil Tyagi[1], Mohammad Aslam Khan[1], Sanjeev Kumar Srivastava[1,2], Ahmed Al-Ghadhban[1], Kari Dugger[3], James E. Carter[4], Seema Singh[1,5]*, Ajay Pratap Singh[1,5]*

***To whom correspondence should be addressed.**

Ajay Pratap Singh, Ph.D.
Professor of Oncologic Sciences
Mitchell Cancer Institute
University of South Alabama
1660 Springhill Avenue
Mobile, AL 36604-1405, U.S.A.
Phone: 251-445-9843; Fax: 251-460-6994
E-mail: asingh@health.southalabama.edu

Seema Singh, Ph.D.
Associate Professor of Oncologic Sciences
Mitchell Cancer Institute
University of South Alabama
1660 Springhill Avenue
Mobile, AL 36604-1405, U.S.A.
Phone: 251-445-9844; Fax: 251-460-6994
E-mail: seemasingh@health.southalabama.edu

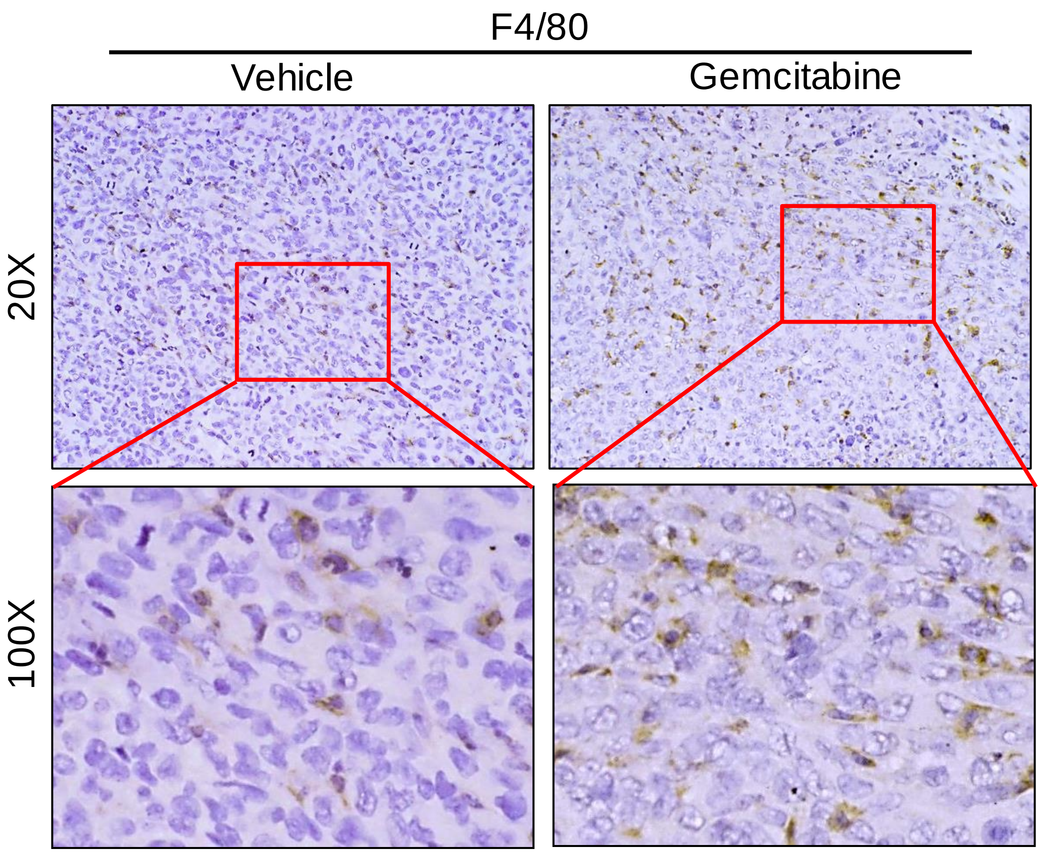

Figure S1. Gemcitabine induces the infiltration of mouse macrophage in pancreatic tumors tissue. Representative images of tumor sections that were stained with F4/80 (mouse macrophage marker). Tumors were resected from mice treated with vehicle or gemcitabine.

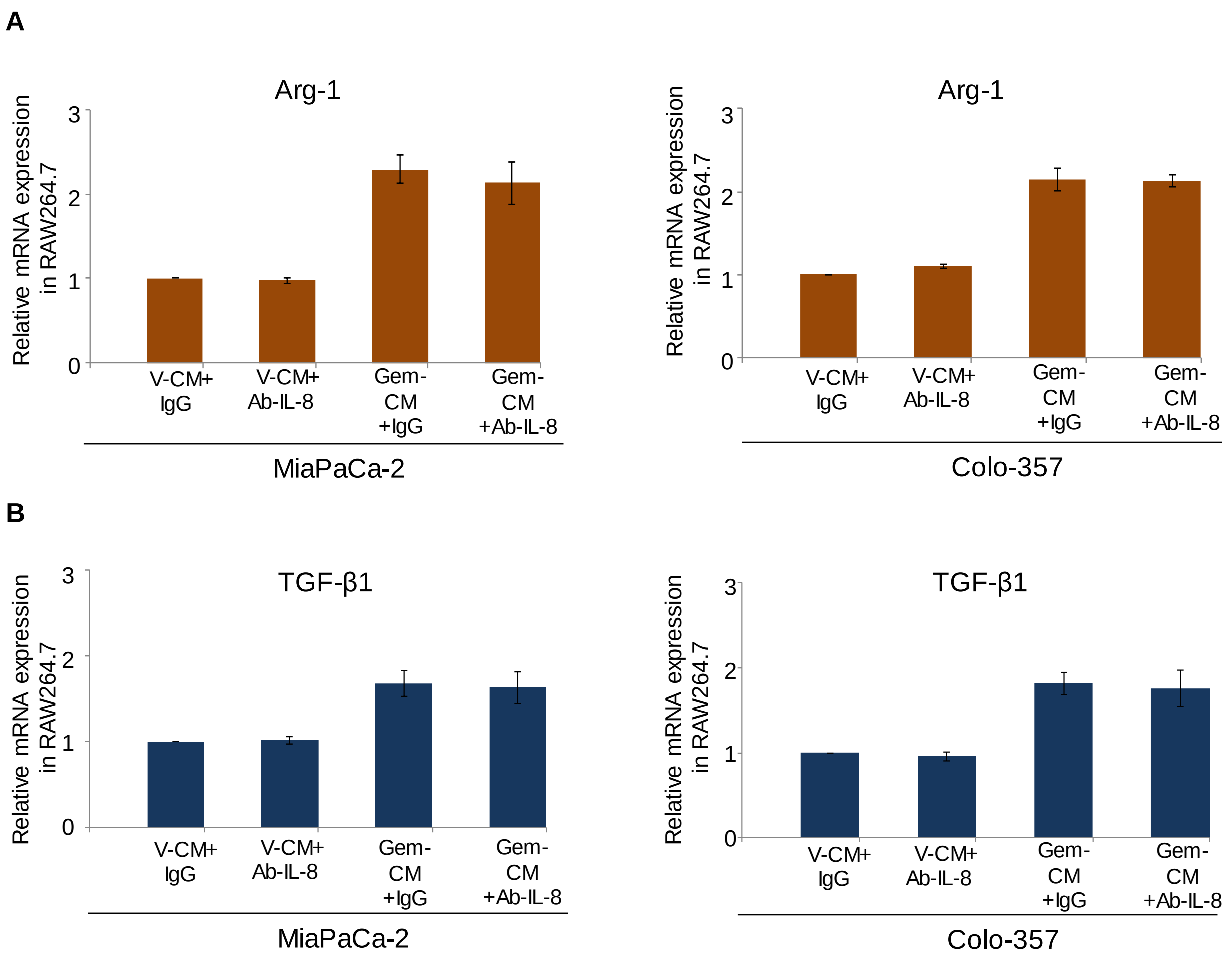

Figure S2. IL-8 neutralization has no effect on the Gem-CM-induced M2 macrophage polarization. RAW264.7 cells were treated with either V-CM or Gem-CM. Both CM were pre-treated with either IL-8 neutralizing antibody or control IgG for 48 h. RNA was isolated, cDNA was prepared, and the expression levels of Arg-1 (A) and TGF-β1 (B), specific markers of M2 macrophage were examined by qRT-PCR. GAPDH was used as internal control. Bars represent mean ±SD.

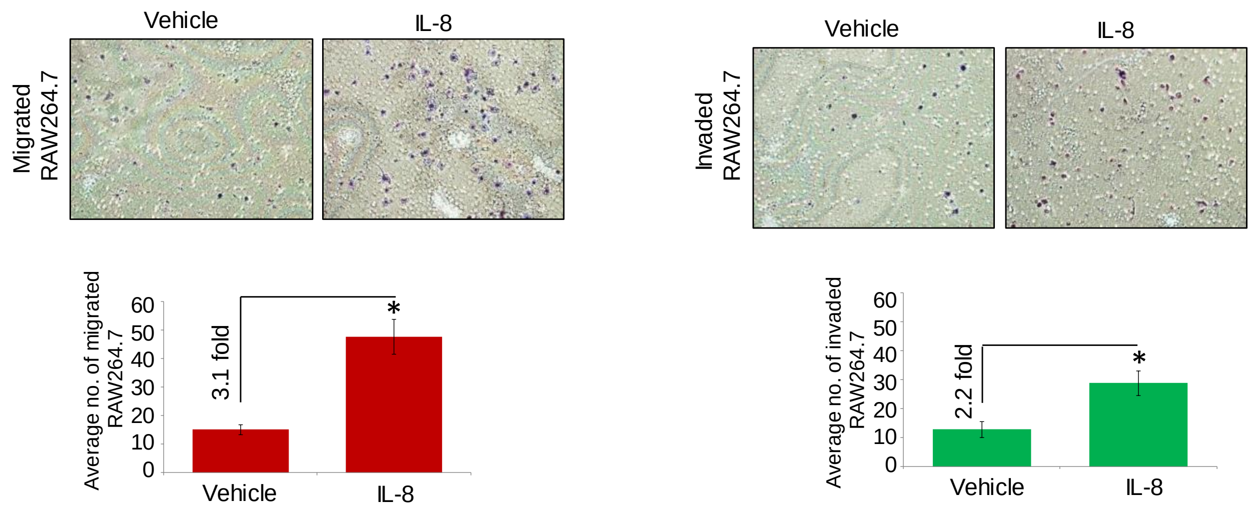

Figure S3. IL-8 promotes infiltration of RAW264.7 macrophages. RAW264.7 cells were seeded on non-coated (for motility assay), or Matrigel-coated (for invasion assay) membranes, recombinant IL-8 was added to the lower chambers to be used as a chemoattractant. Bars represent mean ± SD number of migrated or invaded cells per field. $*p < 0.05$.

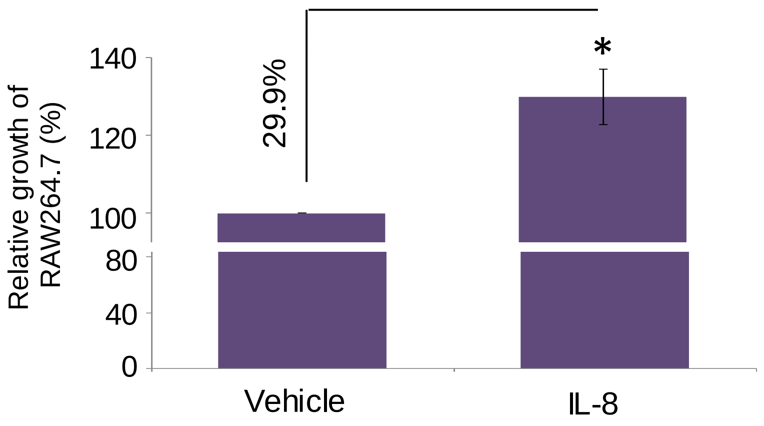

Figure S4. IL-8 induces growth of RAW264.7 macrophages. RAW264.7 cells were seeded into a 96-well plate and treated with either vehicle or recombinant IL-8. Growth was measured by a WST-1 assay after 72 h. Data is presented as mean ± SD; n = 3.*$p < 0.05$.

Table S1: List of oligomers used in this study.

S.No.	Gene Name	Primer Sequence (5′-3′)
1.	*CD45*	Forward: GAACATGCTGCCAATGGTTCT<br>Reverse: TGTCCCACATGACTCCTTTCC
2.	*CD68*	Forward: TTCTGCTGTGGAAATGCAAG<br>Reverse: AGAGGGGCTGGTAGGTTGAT
3.	*Arg-1*	Forward: TTGGGTGGATGCTCACACTG<br>Reverse: TTGCCCATGCAGATTCCC
4.	*CCL3*	Forward: AGTTCTCTGCATCACTTGCTG<br>Reverse: CGGCTTCGCTTGGTTAGGAA
5.	*CCL4*	Forward: CTGTGCTGATCCCAGTGAATC<br>Reverse: TCAGTTCAGTTCCAGGTCATACA
6.	*CCL8*	Forward: TGGAGAGCTACACAAGAATCACC<br>Reverse: TGGTCCAGATGCTTCATGGAA
7.	*IL-20*	Forward: ATGAAAGCCTCTAGTCTTGCCT<br>Reverse: GCCCCGTATCTCAGAAAATCC
8.	*IL-22*	Forward: GCTTGACAAGTCCAACTTCCA<br>Reverse: GCTCACTCATACTGACTCCGT
9.	*CCL5*	Forward: CCAGCAGTCGTCTTTGTCAC<br>Reverse: CTCTGGGTTGGCACACACTT
10.	*IL-8*	Forward: ACTGAGAGTGATTGAGAGTGGAC<br>Reverse: AACCCTCTGCACCCAGTTTTC
11.	*VEGF*	Forward: GAGGAGCAGTTACGGTCTGTG<br>Reverse: TCCTTTCCTTAGCTGACACTTGT
12.	*CSF1*	Forward: TGGCGAGCAGGAGTATCAC<br>Reverse: AGGTCTCCATCTGACTGTCAAT
13.	*CCL2*	Forward: TCTGTGCCTGCTGCTCATAG<br>Reverse: GGGCATTGATTGCATCTGGC
14.	*IL-19*	Forward: ATCCAAGCTAAGGACACCTTCC<br>Reverse: GTCACGCAGCACACATCTAAG
15.	*IL-34*	Forward: CCTGGCTGCGCTATCTTGG<br>Reverse: AGTGTTTCATGTACTGAAGTCGG
16.	*TGF-β1*	Forward: GGCCAGATCCTGTCCAAGC<br>Reverse: GTGGGTTTCCACCATTAGCAC
17.	*TGF-β2*	Forward: CAGCACACTCGATATGGACCA<br>Reverse: CCTCGGGCTCAGGATAGTCT
18.	*IL-4*	Forward: CCAACTGCTTCCCCCTCTG<br>Reverse: TCTGTTACGGTCAACTCGGTG
19.	*IL-13*	Forward: GAGGATGCTGAGCGGATTCTG<br>Reverse: CACCTCGATTTTGGTGTCTCG
20.	*GAPDH*	Forward: ACAACTTTGGTATCGTGGAAGG<br>Reverse: GCCATCACGCCACAGTTTC
